# Prevalence of Vitamin D Deficiency and Its Association with Insulin Resistance in Obese Women with Normal Fasting Glucose

**DOI:** 10.1155/2021/2259711

**Published:** 2021-12-14

**Authors:** Nazish Saleem, Nayab Batool Rizvi, Shan Elahi

**Affiliations:** ^1^School of Chemistry, New Campus, University of the Punjab, Lahore, Pakistan; ^2^Centre for Nuclear Medicine (CENUM), Mayo Hospital, Lahore, Pakistan

## Abstract

**Results:**

A total of 264 obese and 133 normal BMI women (controls) of age range 20-50 years were selected. Obese women had significantly lower vitamin D compared to control women (*P* < 0.05). Among euglycemic (fasting glucose < 100 mg/dl) obese women (*n* = 221), 90 (40.7%) were vitamin D deficient. Serum PTH and calcium levels were negatively correlated, though nonsignificantly with vitamin D (*r* = −0.172, *P* = 0.090, and *r* = −0.051, *P* = 0.557, respectively). The mean age, BMI, waist circumference (WC), hip circumference (HC), waist-to-hip ratio (WHR), fasting glucose, fasting insulin, PTH, and calcium were not significantly different in vitamin D-deficient as compared to nondeficient obese women. IR was detected in 109 (49.3%) obese women. Mean HOMA-IR in vitamin D-deficient women was significantly higher than that in the nondeficient obese women (3.03 ± 1.64 vs. 2.40 ± 1.02; *P* = 0.041), but the percentage of women with IR was comparable in both groups (51.1% vs. 45.8%; *P* = 0.745). Univariate analysis revealed that HOMA-IR was negatively correlated with vitamin D and positively with BMI and PTH. A multivariate regression analysis, stepwise method revealed that BMI and PTH were independent determinants of HOMA-IR instead of vitamin D.

**Conclusion:**

More than 40% of obese women were vitamin D deficient. Among euglycemic obese women, 49% were insulin resistant. Prevalence of insulin resistance, though negatively correlated with vitamin D, could be better explained by BMI and PTH levels.

## 1. Introduction

Obesity is associated with a number of noncommunicable chronic diseases such as type 2 diabetes, coronary disease, chronic kidney disease, and asthma [1]. Adipose tissue is considered an endocrine organ that regulates many physiological processes. Inflammation of adipose tissue is associated with disrupted metabolic homeostasis, insulin resistance (IR), and type 2 diabetes [2]. Obesity and diabetes share a common IR pathway and contribute to chronic hyperglycemia [2, 3]. IR affects glucose homeostasis and pancreatic beta-cell functioning and is an indicator for developing of hypertension, dyslipidaemia, and type 2 diabetes [3].

Vitamin D has recently been associated with several of the contributing factors known to be linked to the development of IR and hence type 2 diabetes mellitus including defects in pancreatic beta cell function, insulin sensitivity, and systemic inflammation [4, 5]. Vitamin D deficiency (VDD) has been described as a risk factor for type 2 diabetes in obese humans [4]. The deposition of vitamin D in fat tissues can result in lower vitamin D bioavailability in obese subjects [6]. Recently, several studies have reported a significant association between high circulating levels of 25(OH)D and lower incidence of type 2 diabetes [7, 8]. However, no such association between vitamin D and type 2 diabetes was observed in other studies [9–10]. Many reasons for this discrepancy have been proposed [10]. An overlooked reason may be the nonconsideration of concomitant vitamin D and its related metabolites, i.e., parathyroid hormone (PTH) and calcium in elucidating the relation between vitamin D and glucose metabolism. Vitamin D is crucial for sufficient calcium absorption through PTH regulation. Vitamin D deficiency and increased concentration of PTH [11] as well as calcium [12] are implicated in the augmented risk of diabetes. This is especially relevant to Pakistani subjects as they are reported to have low vitamin D, high PTH, and increased calcium level with nonconventional relation between vitamin D, PTH, and calcium [13, 14].

Moreover, most studies regarding vitamin D and glucose metabolism are conducted in obese subjects without prior stratification based on the glycemic status. A few studies have explored the association between vitamin D and insulin resistance in obese and general population with normal fasting glucose levels [15–17]. This study is conducted to know the association between vitamin D and its related metabolites with IR in obese women with normal fasting glucose.

## 2. Patients and Methods

### 2.1. Study Participants

The study was conducted during the year 2019-2020 at the Centre for Nuclear Medicine (CENUM), Mayo Hospital Lahore. The institutional ethics committee (Bio-Ethical Committee, Institute of Chemistry, Punjab University, Lahore) approved the study. Obese women (BMI ≥ 30 kg/m^2^) with age range 20-50 years from Mayo Hospital Lahore and Institute of Chemistry, Punjab University, Lahore, were registered. A standardized questionnaire was filled by all the participants including their personal information, medical history, family history of obesity, family history of DM, presence of any chronic disease, and use of vitamin D supplements. All anthropometric information containing age, weight, height, body mass index (BMI), waist circumference (WC), and hip circumference (HC) was recorded. The exclusion criteria were (i) an inability or unwillingness to participate in the study, (ii) intake of vitamin D supplementation, (iii) use of medications that could potentially interfere with vitamin D metabolism, and (iv) presence of any concomitant clinical disease that could influence vitamin D metabolism (e.g., renal, hepatic, other endocrinological disorders and malignancies).

Nonobese women (BMI: 18-24.9 kg/m^2^) were randomly selected as a reference population for comparative purposes (control). They were healthy, nonpregnant women having no history of vitamin D supplementation with age range 20-50 years.

### 2.2. Anthropometric Measurements

All anthropometric measurements were taken when subjects were in fasting. Weight was measured when the participants had light clothes and wearing no shoes with the help of a digital scale nearest to 0.1 kg. For height measurement, a stadiometer was used nearest to 0.1 cm. Body mass index was calculated by dividing the weight with the square of height, kg/m^2^. Waist and hip circumference was measured with the help of a tape nearest to 0.1 cm. For waist measurement, the smallest diameter of the waist was measured, while for hip circumference, the largest diameter of the hips was noted.

### 2.3. Laboratory Investigations

Venus blood sample was collected of all the participants after an overnight fast. Blood was placed for an hour, and after that, centrifugation of samples was done at 2000 rpm, and the serum was transferred to microtubes and kept at -20°C until determination of fasting glucose, fasting insulin, serum 25(OH)D, PTH, and calcium concentration. A spectrophotometer was used for fasting glucose determination and PTH, and fasting serum insulin was assessed by immunoradiometric assay (IRMA) and vitamin D by radioimmunoassay (RIA) method. Obese women with fasting glucose level < 100 mg/dl were considered normal (euglycemic). Serum vitamin D levels less than 12 ng/ml and more than 30 ng/ml were considered as deficient and sufficient, respectively, according to recent recommendation of the Third International Conference on Controversies in Vitamin D held in Gubbio, Italy, on September 10–13, 2019 [18]. Homeostasis model assessment of insulin resistance (HOMA-IR) was used for evaluation of IR by the following formula [16]:

(1) $$\begin{array}{c} \text{HOMA}‐\text{IR}=\frac{\text{Fasting Insulin }\left(\mu \text{IU}/\text{ml}\right)\times \text{Fasting Glucose }\left(\text{mg}/\text{dl}\right)}{405}. \end{array}$$

### 2.4. Data Analysis

The analysis of data was carried out using Microsoft SPSS program (SPSS 16.0, IBM). Continuous variables were presented as mean (±SD), and categorical variables were presented as absolute numbers and percentage. Before conducting statistical analysis, data were checked for normal distribution. In case of skewed distribution, median was used or data was log transformed to normalize the distribution. Student *T* test was used to compare mean values, and the chi-square test was applied to test the significance of difference between two arbitrary groups. Insulin resistance was considered a dependent outcome of obesity indices (BMI, WC, HC, WHR) as well as vitamin D and its related biochemical parameters (PTH and calcium). Their mutual relation was determined by enumeration of univariate correlation as well as multiple regression analysis. A *P* value of 0.05 was considered significant.

## 3. Results

At CENUM, 264 obese (BMI > 30 kg/m^2^; mean 34.3 ± 5.3 kg/m^2^) and 133 nonobese (BMI 18-24.9 kg/m^2^; mean 23.4 ± 2.6 kg/m^2^) were selected for this study.  Table 1 shows the comparison of mean age, BMI, vitamin D, and vitamin D-related metabolites in both groups of women. Both groups had comparable age and calcium level (both *P* > 0.05). However, obese women had a significantly lower mean level of vitamin D; a higher percentage of women with vitamin D deficiency increased the mean level of PTH compared to nonobese women (all *P* < 0.05).

Among the obese group of women, 221 women (83.7%) had a fasting glucose level < 100 mg/dl (euglycemic). Further analysis was carried out in these euglycemic obese women. Their mean BMI, fasting glucose, fasting insulin, and HOMA-IR values were 35.5 ± 4.6 kg/m^2^ (range: 30.0–49 kg/m^2^), 88.8 ± 8.9 mg/dl (range: 57-99 mg/dl), 12.5 ± 6.3 *μ*IU/ml (range: 2.1–36.1 *μ*IU/ml), and 2.77 ± 1.43 (range: 0.66-7.83), respectively. The HOMA − IR ≥ 2.5 (insulin resistance) was detected in 109 (49.3%) women. Vitamin D levels were widely scattered ranging from 3.0 to 67 mg/dl, and 90 (40.7%) women were vitamin D deficient according to the latest cutoff level for vitamin D deficiency (<12.0 ng/ml). The median concentration of vitamin D was 13.87 (interquartile range 10.0, 18.74). The corresponding figures for serum PTH and calcium were 21.5 pg/ml (13.3, 33.3 pg/ml) and 9.15 mg/dl (8.48, 9.65 mg/dl), respectively. Serum PTH and calcium levels were negatively correlated with vitamin D, but these correlations were not statistically significant (*r* = −0.172, *P* = 0.090, and *r* = −0.051, *P* = 0.557, respectively). However, mutual correlation between PTH and calcium was negative and significant (*r* = −0.267, *P* = 0.008).

Table 2 presents the comparison of anthropometric and biochemical characteristics of vitamin D-deficient and nondeficient obese women. The mean anthropometric variables (age, BMI, WC, HC, and WHR) were comparable in both groups. Similarly, mean serum fasting glucose, fasting insulin, PTH, and calcium were not significantly different in vitamin D-deficient as compared to nondeficient obese women. However, mean HOMA-IR in vitamin D-deficient women was significantly higher than that in the nondeficient obese women (2.87 ± 1.41 vs. 2.40 ± 1.02; *P* = 0.041), but the percentage of women with HOMA − IR ≥ 2.5 was comparable in both groups (51.1% vs. 45.8%; *P* = 0.7449).

Table 3 shows univariate analysis of HOMA-IR with anthropometrics as well as biochemical parameters. Results revealed that HOMA-IR was not significantly related with age, WC, HC, WHR, and calcium. It was significantly correlated with BMI, vitamin D, and PTH. The magnitude of correlation between HOMA-IR and these variables was almost the same. However, HOMA-IR was negatively correlated with vitamin D and positively with BMI and PTH. A multiple regression analysis and stepwise method was performed with HOMA-IR as the outcome and BMI, vitamin D, and PTH as significant predictors. This is shown in Table 4. Instead of vitamin D, both BMI and PTH were found independent determinants of HOMA-IR.

## 4. Discussion

This study was planned to assess the current prevalence of vitamin D deficiency in obese women as compared to nonobese women and to delineate its relation with development of IR in euglycemic obese women. According to our results, obese women were relatively more vitamin D deficient as compared to normal women. This is in accordance to two small studies carried out in local obese women [19, 20]. These studies have used 20 ng/ml as the cutoff limit to define vitamin D deficiency, but we have used the latest cutoff level (12 ng/ml) for this purpose [18]. In spite of the relatively lower cutoff value, our percentage of vitamin D-deficient obese women (40.7%) was comparable to that of one of the above mentioned studies, i.e., 40% [19]. As a consequence of relatively more vitamin D deficiency in obese women, the mean PTH concentration was significantly higher than in nonobese women (20.8 ± 15.9 versus 17.1 ± 12.7, respectively, *P* = 0.0034), but the mean serum calcium level was comparable in both groups. We found no such study in Pakistani obese women to verify these findings. However, this observation is in line with a study conducted in Norway that reported low vitamin D, high PTH, and increased calcium level in immigrant subjects from Pakistani with mean BMI of 28.1 kg/m^2^ [13]. The reason for higher calcium in the presence of high PTH was unclear according to the Norwegian study [13]. In our study, we found that among euglycemic obese women, though PTH and calcium levels were negatively correlated significantly (*r* = −0.267, *P* = 0.008), their individual negative correlation with vitamin D was not significant. Such noncustomary relation between vitamin D, PTH, and calcium is also reported among Pakistani women in other studies [13, 14].

It has been demonstrated that diabetes mellitus may develop from vitamin D deficiency acting directly and indirectly [4, 5]. Direct action includes impaired pancreatic beta-cell dysfunction and thus decreased insulin sensitivity or enhanced insulin resistance. Due to the presence of both 1-*α*-hydroxylase and vitamin D receptor (VDR) in pancreatic *β* cells, vitamin D is important for insulin synthesis and release [5, 21]. Previous studies suggest that vitamin D deficiency is associated with a proinflammatory state in obesity [4, 5]. Indirect causes of developing diabetes mellitus may be increased inflammation as well as enhanced calcemic hormones like PTH especially in obese and vitamin D-deficient subjects [21]. Our results are in accordance to this notion as not only low vitamin D but also increased PTH had direct bearing on IR in our obese women. We found a close correlation of vitamin D with IR in euglycemic obese women. Besides vitamin D, serum PTH was also positively correlated with IR. Thus, it seems that instead of vitamin D alone, concurrent vitamin D deficiency along with higher serum PTH levels was more closely linked to IR in obese women. This proves that among vitamin D-deficient obese women, those with a higher PTH level are at increased risk of developing insulin resistance. This observation is in accordance with Chiu et al. who reported that the first-phase insulin response was positively correlated with plasma intact PTH level that was inversely correlated with insulin sensitivity index in healthy subjects with glucose tolerance [11]. Similarly, Stanley et al. reported that vitamin D was not associated with glucose homeostasis but PTH was positively correlated with a quantitative insulin sensitivity check index in obese girls and had negative association with HOMA-IR [22].

The probable mechanism by which PTH affects insulin sensitivity may be calcium haemostasis in obese individuals [23]. Hultin et al. found that obese subjects had impaired calcium metabolism due to the left-shifted relation of PTH with ionized calcium that has a role in the development of secondary hyperparathyroidism [24] that may reduce insulin sensitivity [25]. Moreover, Chang et al. in an in vitro study found that the differentiated 3T3-L1 adipocyte treatment with PTH suppressed insulin signalling through the cAMP pathway and insulin-stimulated glucose uptake via insulin receptor substrate-1 (IRS-1) phosphorylation [26]. This is supported by a study conducted by Karras et al. who evaluated the combined effect of vitamin D and the parathyroid hormone on glucose dysregulation and insulin resistance in subjects with prediabetes and compared them with the healthy controls. They concluded that the PTH could affect the glucose haemostasis independently to vitamin D concentration in prediabetic patients [27].

This study has important implications regarding DM prevention in obese women. Recently, in a meta-analysis of randomized clinical trials, Lotito et al. had suggested that obesity is often associated with vitamin D deficiency as well as secondary hyperparathyroidism, and vitamin D supplementation typically leads to the reductions in PTH levels [28]. It implies that improvement of the vitamin D status can suppress the PTH that may ameliorate insulin resistance in obese subjects. This strategy of vitamin D supplementation to prevent DM in obese subjects could be an alternative to conventional preventive measures. Other treatment options are also required to be worked out by further investigation in this field.

## Figures and Tables

**Table 1:** Comparison of anthropometric, vitamin D, and vitamin D-related metabolites in obese and control women.

Parameters (units)	Obese	Control	*P* value
No.	264	133	—
Age (years)	33.0 ± 8.4	31.6 ± 10.9	0.2182
BMI (kg/m^2^)	34.3 ± 5.3	23.4 ± 2.6	≤0.001
VD (ng/ml)	16.0 ± 10.6	20.1 ± 13.8	0.0051
VD < 12 [*n* (%)]	107 (40.5)	36 (27.1)	0.0292
PTH (pg/ml)	20.8 ± 15.9	17.1 ± 12.7	0.0034
Ca (mg/dl)	9.1 ± 1.0	9.2 ± 0.9	0.2065

BMI: body mass index; VD: vitamin D; PTH: parathyroid hormone; Ca: calcium.

**Table 2:** Comparison of anthropometric and biochemical parameters of euglycemic obese women (*n* = 221) according to the serum vitamin D level.

Parameters (units)	VD level		*P* value
	<12 ng/ml	≥12.0 ng/ml	
No.	90	131	—
Age (years)	34.1 ± 8.0	34.6 ± 8.6	0.802
BMI (kg/m^2^)	35.3 ± 4.8	36.2 ± 5.6	0.241
WC (cm)	107 ± 10.5	106 ± 11.8	0.802
HC (cm)	116 ± 10.4	116 ± 9.7	0.982
WHR	0.93 ± 0.06	0.92 ± 0.08	0.703
FG (mg/dl)	91.8 ± 22.7	93.9 ± 17.5	0.445
FI (*μ*IU/ml)	10.8 ± 2.5	11.7 ± 2.0	0.436
HOMA-IR	3.03 ± 1.64	2.40 ± 1.02	0.041
HOMA − IR ≥ 2.5 [*n* (%)]	49 (51.1)	60 (45.8)	0.745
PTH (pg/ml)	22.7 ± 13.6	24.0 ± 12.9	0.662
Ca (mg/dl)	9.3 ± 1.0	9.0 ± 1.0	0.434

VD: vitamin D; BMI: body mass index; WC: waist circumference; HC: hip circumference; WHR: waist-to-hip ratio; FG: fasting glucose; FI: fasting insulin; HOMA-IR: homeostasis model for assessment of insulin resistance; PTH: parathyroid hormone; Ca: calcium.

**Table 3:** Univariate correlations of HOMA-IR with anthropometrics and biochemical parameters in obese women.

Parameters (units)	*r*	*P* value
Age (year)	0.065	0.450
BMI (kg/m^2^)	0.218	0.011
WC (cm)	0.182	0.068
HC (cm)	0.163	0.102
WHR	0.069	0.491
VD (ng/ml)	-0.227	0.008
PTH (pg/ml)	0.224	0.027
Ca (mg/dl)	-0.132	0.125

BMI: body mass index; WC: waist circumference; HC: hip circumference; WHR: waist-to hip-ratio; VD: vitamin D; PTH: parathyroid hormone; Ca: calcium.

**Table 4:** Multivariate regression analysis, stepwise method of HOMA-IR (outcome) and its predictors (BMI, vitamin D, and PTH) in obese women.

Models	*β*	SE	95% CI		*P*
			Lower	Upper	
*Model 1*					
BMI	0.092	0.031	0.031	0.153	0.004
*Model 2*					
BMI	0.088	0.030	0.028	0.148	0.005
PTH	0.017	0.008	0.001	0.034	0.035

BMI: body mass index; PTH: parathyroid hormone; OR: odds ratio; SE: standard error.

## Data Availability

The data used to support the findings of this study are included in the article.
